# Internet‐based support program on parenting outcomes for Chinese primiparous women: Study protocol for a randomized controlled trial

**DOI:** 10.1111/jan.14517

**Published:** 2020-09-09

**Authors:** Xujuan Zheng, Lingling Huang, Qiyu Fang, Yan Zhang, Yao Zhang, Xilin Li, Ziwen Ye, Qun Wang

**Affiliations:** ^1^ Health Science Centre Shenzhen University Shenzhen Guangdong China

**Keywords:** internet‐based intervention, maternal self‐efficacy, nursing, postpartum depression, primiparous women, social support

## Abstract

**Aim:**

To evaluate the effects of internet‐based support program for primiparous women in terms of improving the levels of maternal self‐efficacy, social support, and satisfaction; and reducing their postpartum depression symptoms.

**Design:**

A single‐blinded, multicentre, randomized, controlled, parallel‐group pre‐test and repeated post‐test design.

**Methods:**

Based on the self‐efficacy theory and the social exchange theory, the internet‐based support program has five modules: (a) learning forum of parenting knowledge and skills; (b) communication forum; (c) ask‐the‐expert forum; (d) baby home forum; and (e) reminder forum. Primiparous women will be recruited in the obstetric wards of two university‐affiliated hospitals in China. The participants (*N* = 258) will be randomly allocated to the intervention group that receive routine care and access to the internet‐based support program and the control group that receive routine care during the 3 months postpartum. Maternal self‐efficacy, social support, and postpartum depression symptoms will be measured at baseline, immediately after the intervention (post‐test 1) and 3 months after the intervention (post‐test 2). The study was funded in January 2018 and was ethically approved in May 2020.

**Discussion:**

If the internet‐based support program has positive outcomes, it will contribute to the scientific and practical knowledge of nursing interventions to support primiparous women on parenting; and could become the routine health care for health professionals to enhance parenting ability and mental well‐being of new mothers.

**Impact:**

As the first RCT study on parenting outcomes using a rigorous research design and a theoretical framework in China, this research will contribute to evidence on the effectiveness of using internet platform to support women after childbirth. The results could help to advance research about the use of internet‐based intervention methods to improve women's maternal self‐efficacy, social support, satisfaction, and to alleviate depression symptoms.

Chinese Clinical Trial Registry: ChiCTR2000033154

## 摘要

**目标:**

评价基于互联网的支持计划对初产妇女在提高母亲自我效能感、社会支持和满意度、减轻产后抑郁症状方面的效果。

**设计:**

单盲、多中心、随机、对照、平行组试验前和重复试验后设计。

**方法:**

基于自我效能感理论和社会交换理论,基于互联网的支持计划分为五个模块:(a)育儿知识和技能学习论坛;(b)交流论坛;(c)专家咨询论坛;(d)婴儿之家论坛;(e)注意事项提示论坛。中国两所大学附属医院的产科病房将招募初产妇女。参与者(N=258)将被随机分配到接受常规护理和基于互联网的支持计划的干预组和产后3个月接受常规护理的对照组。在基准线位置、干预后(试验后1)和干预后3个月(试验后2)测量母亲的自我效能感、社会支持和产后抑郁症状。该研究于2018年1月获得资助,并于2020年5月获得道德批准。

**讨论:**

如果基于互联网的支持计划取得积极成果,它将有助于提高护理干预措施的科学性和实用性,以支持初产妇女的育儿技能;并可能成为保健专业人员的常规保健,以提高新妈妈的育儿能力和心理健康。

**影响:**

作为中国第一个运用严谨研究设计和理论框架开展的育儿效果随机对照研究,本研究将有助于证明利用互联网平台支持产后妇女的有效性。本研究结果有助于推动基于互联网的干预方法提高女性作为母亲的自我效能感、社会支持、满意度以及缓解抑郁症状的研究。

中国临床试验注册中心:ChiCTR2000033154

## 1 INTRODUCTION

Primiparous women usually confronted with many parenting problems in the early motherhood, which had a negative impact on the well‐being of mothers and infants (Zheng, Morrell, & Watts, 2015, 2018a). As an important predictor of parenting (Li & Liu, 2019), maternal self‐efficacy (*MSE*) is the belief of a mother holds of her capability about the organization and performance of parenting tasks (Montigny & Lacharite, 2005). Researchers found that the samples of women in the UK (Whittaker & Cowley, 2012), USA (Fulton, Mastergeorge, Steele, & Hansen, 2012), Finland (Salonen et al., 2009), and Canada (Pierce et al., 2010) had a high level of *MSE*; however, Chinese primiparous women had a moderate level of *MSE* and especially were less sure of their capability in recognition and management of some common diseases and emergency care for their infants (Zheng, Morrell, & Watts, 2018b).

Many factors are reported to affect *MSE* and the two main factors relating to *MSE* are postpartum depression (PPD) and social support (Zheng, Morrell, & Watts, 2018a). Evidences demonstrated that a greater proportion of Chinese primiparous women had PPD than women in Western countries, owing to the high expectations of being mothers and the sensitive and stressful relationship with their mother‐in‐law in Chinese culture (Gao, Chan, & Sun, 2012; Zheng et al., 2018b). The literature consistently showed that social support has a positive impact on *MSE* in various countries (Leahy‐Warren & McCarthy, 2011; Shorey, Chan, Chong, & He, 2015a). Studies, for example, by Zheng et al. (2018b) suggested that Chinese primiparous women receive inadequate support from health professionals, and they experienced a lack of informational and appraisal support on various newborn‐care tasks.

To enhance the parenting outcomes, some interventions were conducted for new mothers. For example, a randomized controlled trial (RCT) in Spain assessed the outcomes of a mindfulness‐based intervention on maternal psychological distress, well‐being, and *MSE* in breast‐feeding mothers (Perez‐Blasco, Viguer, & Rodrigo, 2013). The intervention was carried out over the meditation course of 8 weeks at a rate of one 2‐hr session per week. Compared with the control group, mothers in the treatment group had significantly higher *MSE* scores (*F* = 10.83, *p* = .004) from pre‐test of 78.85 (*SD* 9.00) to post‐test of 88.92 (*SD* 6.71). Moreover, mothers who received the treatment exhibited significantly less anxiety, stress, and psychological distress. In mainland of China (Gao et al., 2012; Gao, Sun, & Chan, 2014), a RCT was to examine the effects of interpersonal psychotherapy‐oriented childbirth education program consisted of two 90‐min group education sessions and one telephone follow up within 2 weeks after childbirth. The study group had a significantly higher level of social support (65.92 *SD* 8.10) (*t* = 2.33, *p* = .021), *MSE* (37.00 ± 5.13) (t = 2.43, *p* = .016), and less depressive symptoms (5.61 *SD* 3.33) (t = 2.39, *p* = .018) at 3 months postpartum when compared with the control group (their corresponding scores were 63.11 *SD* 8.67; 35.21 *SD* 5.14; 6.87 *SD* 3.97).

Evidence suggested that the traditional face‐to‐face interventions of parenting did have an effect; however, the feasibility and generalizability of traditional interventions were challenged by the huge number of primiparous women, the insufficient financial commitment to health care from the government (5.5% of the GDP), and the shortage of health professionals in China (Zhu, Ebert, Liu, & Chan, 2017). Fortunately, the development of E‐health and M‐health technologies provides alternatives to promote interaction between health professionals and patients outside the clinic (Mobasheri et al., 2014). Due to its convenience and increased accessibility, internet has potential to provide a promising platform for medical interventions (Zhu et al., 2017). In 2019, 61% of the Chinese population used the internet via mobile or computer devices (China News, 2020). The support program based on internet could provide an innovative and easily accessible intervention approach that can reach larger groups of women (Van den Berg, Gielissen, Ottevanger, & Prins, 2012). In Finland, a quasi‐experimental design was used to evaluate the effectiveness of an internet‐based intervention to support maternal satisfaction and *MSE* (Salonen et al., 2011). To the best of our knowledge, no RCTs by internet have been conducted in China on parenting outcomes for primiparous women.

## 2 BACKGROUD

Transition to motherhood will bring various changes to the lives of women, who need to gain parenting knowledge and skills, adjust to the new household relationship, and fulfil self‐expectations as mothers (Salonen et al., 2011). Many women find it difficult to manage these physical, social, and psychological challenges in the early motherhood (Kunseler, Willemen, Oosterman, & Schuengel, 2014; Law et al., 2019); especially for the first‐time mothers owing to the lack of previous parenting experience (Leahy‐Warren & McCarthy, 2011).

The traditional face‐to‐face interventions, such as lecture and home visiting, were proved to be effective in the improvement of parenting outcomes; however, the feasibility and generalizability of these traditional interventions methods were challenged by the huge number of primiparous women and the shortage of health professionals in China. Thus, the alternative, innovative, and easily accessible intervention approach need to be designed. Evidence showed that internet interventions can contain more channels of media to tailor information, reach larger groups of women, and can provide more anonymity compared with face‐to‐face interventions (Van den Berg et al., 2012). Therefore, the internet‐based support program (ISP) for Chinese primiparous women will be conducted to improve their parenting ability, social support, and well‐being.

The theoretical framework of ISP was based on the Bandura's self‐efficacy theory (Bandura, 1997) and the social exchange theory (House, 1981). Bandura (1997, p.3) defined self‐efficacy as “a belief in one's abilities to organize and execute the course of action required to attain a goal or perform a certain task” and believed that self‐efficacy affected the regulation and maintenance of behaviour. Four major elements were identified that affected self‐efficacy: (a) previous experience; (b) vicarious experience; (c) verbal persuasion; and (d) physiological and emotional states (Bandura, 1997). Previous experiences involve previous personal accomplishments and successes. Vicarious experiences are gained by watching others achieve success in similar situations. Verbal persuasion comes from verbal cues and feedback from others. Physiological and emotional states are defined as a person's perception of his/her physical and mental state.

Social support involves both the perception of available assistance and satisfaction with received support (Tietjen & Bradley, 1985); and could be conceptualized by structural and functional components (House, 1981). The structural social support has been conceived as social network, which may be informal (family members, friends) or formal (health professionals), depending on the relationship with its recipient. The functional social support has been conceived as informational, instrumental, emotional, and appraisal support (House, 1981).

**Figure 1:**
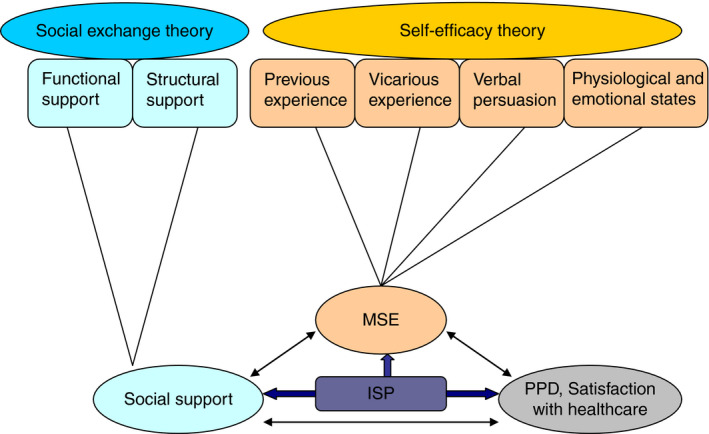
The theoretical framework of the ISP adapted from Shorey et al. (2015b). Note: *MSE*, Maternal self‐efficacy; PPD, Postpartum depression; ISP, Internet‐based support program [Colour figure can be viewed at wileyonlinelibrary.com]

It is said that the appropriate levels of self‐efficacy and social support are essential components of interventions to improve maternal parenting outcomes (Shorey, Chan, Chong, & He, 2015b). The ISP aims to enhance *MSE* and social support to promote mothers’ ability to complete various parenting tasks, thus, improving their psychological well‐being. The multicomponent intervention of ISP program includes a learning forum, a communication forum, an ask‐the‐expert forum, a baby home forum, and a reminder forum that derived from the four factors of the self‐efficacy theory and the functional and structural social support concepts of the social exchange theory. For example, in terms of self‐efficacy theory, the previous experiences involve the provision of parenting knowledge and skills in the learning forum and reminder forum. Vicarious experiences include the parenting techniques taught through multimedia resources in the learning forum, and the sharing parenting experience and the feeling of motherhood transition in the communication forum and baby home forum. Verbal persuasion comes from feedback and suggestions from health professionals and other mothers in the ask‐the‐expert forum and communication forum. The above learning materials, the sharing of experiences, and the various supports from health professionals and other mothers may modify women's physiological and emotional states. According to the social exchange theory, the structural social networks of women are built and the various kinds of functional supports are provided from the health professionals and others through the designed communication forum, ask‐the‐expert forum, and baby home forum. The theoretical framework of the ISP is shown in Figure 1.

## 3 THE STUDY

### 3.1 Aim

This study aims to evaluate the effects of ISP for Chinese primiparous women in terms of improving the levels of *MSE*, social support, and satisfaction; and reducing their postpartum depression symptoms.

The study hypothesizes that compared with the control group at baseline, post‐test 1, and post‐test 2, women in the intervention group will report statistically significant:

1. Improved *MSE* and social support;
2. Reduced postpartum depression symptoms; and.
3. Great satisfaction with healthcare received after childbirth.

### 3.2 Design

#### 3.2.1 Trial design

**Figure 2:**
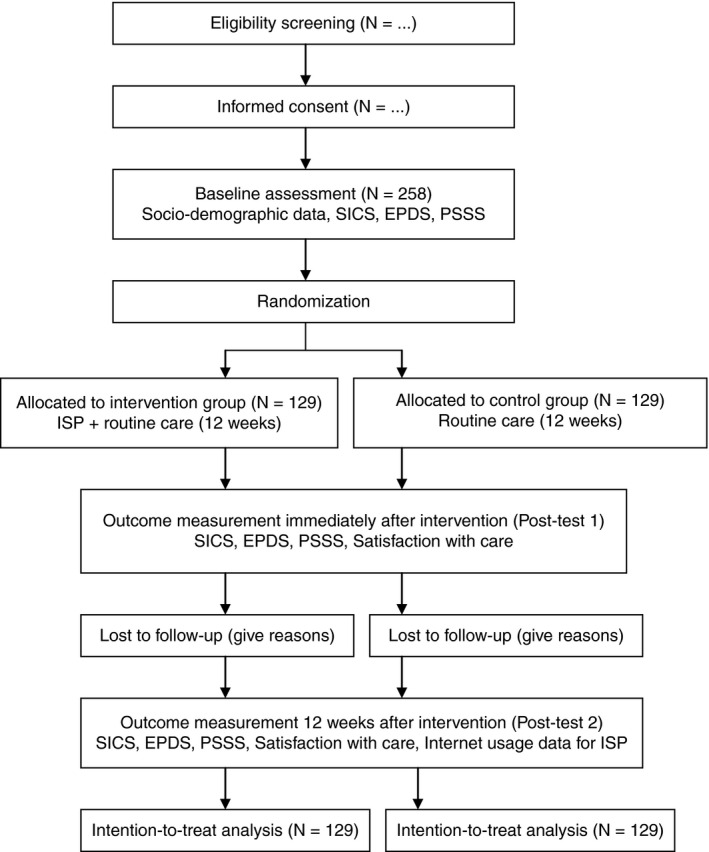
CONSORT flowchart of the study adapted from Schulz et al. (2011). Note: SICS: the Self‐efficacy in Infant Care Scale; EPDS: the Edinburgh Postnatal Depression Scale; PSSS: the Postpartum Social Support Scale

A single‐blinded, multicentre, randomized, controlled, parallel‐group pre‐test, and repeated post‐test design will be conducted to investigate the effect of the ISP for Chinese primiparous women in the initial postpartum period. The study follows the SPIRIT 2013 Statement and the guidelines for the Standard Protocol of Clinical Trials (Chan et al., 2013). The Consolidated Standards of Reporting Trials (CONSORT) (Schulz, 2011) flowchart is presented in Figure 2.

#### 3.2.2 Study setting and participants

The research will be carried out in the two university‐affiliated tertiary public hospitals in China, where the live births in each study hospital are over 2,000 babies per year. The inclusion criteria will be: first‐time mothers with healthy babies; aged 18 years or above; married; and able to response the questionnaires. Exclusion criteria will be: women or their children have severe physical and mental diseases.

Sample size will be estimated based on previous research with a medium to small effect size (Cohen, 1992). With a power of 0.80, an alpha set at 0.05, and an effect size of 0.35 for the primary outcome of PPD (EPDS scores), each group will be 90 women (Gao et al., 2012). We estimate a 30% attrition rate based on an attrition rate of 9%–29% in previous research (Gao et al., 2012). A minimum of 258 women (129 in each group) are required.

#### 3.2.3 Recruitment

Participants will be recruited when women are admitted in the obstetric wards of hospitals. The obstetric nurses will introduce the ISP to women who meet the inclusion criteria. Posters and leaflets about the program also will be strategically distributed in the obstetric wards to inform all women and their family members. Women who verbally agree to participants will be approached by the researchers to provide them with an information sheet and to answer their any questions about the research. In this approach, women were not asked to give consent as it was considered important to make sure that women had enough time (at least 1 day) to read the information sheet, think about participation, and discuss it with their family members. The researchers will acquire participants’ written informed consent on the next day.

The recruitment period is expected to extend over a 3‐month period. Data will be collected before randomization (baseline), immediately after the intervention (post‐test 1), and 3 months after the intervention (post‐test 2). Recruitment of participants began in May 2020. Primary endpoints (baseline and post‐test 1) and follow‐up measure (post‐test 2) will be expected to be completed in January 2021.

#### 3.2.4 Randomization and blinding

The Research Randomizer will be used to randomly assign participants into the experimental group and the control group with a distribution ratio of 1:1. The allocation sequence will be put into the opaque and sealed envelopes. Blinding will not be possible for the researchers during all the research process; however, the group allocation will be masked during the recruitment until the baseline measures are completed. In terms of trial participants, outcome assessors, and data analysts, they will be blinded to the participants’ group allocation.

#### 3.2.5 Intervention

The women in the control group will receive routine care. Routine care consists of supports from the obstetricians and obstetric nurses during the 3–5 days hospitalization; and home visits from the community doctors on the 3rd, 7th, 14th, and 28th days postpartum (Zheng, Morrell, & Watts, 2015). The women in the study group will have access to the ISP and receive routine care in the postpartum period. The details of intervention are described in Table 1.

**Table 1:** The details of intervention

	Intervention Group	Control Group
Service	ISP + routine care	Routine care
Provider	Researchers	Obstetricians, obstetric nurses, community doctors
Place	Web environment	Hospital, home
Form	Internet	Face‐to‐face
Contents	Learning forum Communication forum Ask‐the‐expert forum Baby home forum Reminder forum	Routine postpartum home visiting
Duration and frequency	From childbirth to 3‐month postpartum The total intervention time not less than 12 weeks Reminder telephones every week, reminding them to log in the ISP at least twice a week, and no less than total 1 hr per week	From childbirth to 1 month postpartum At least two home visits
Assessment	Baseline (pre‐intervention) Post‐test 1 (immediately after intervention) Post‐test 2 (3 months after intervention)	Baseline (pre‐intervention) Post‐test 1 (immediately after intervention) Post‐test 2 (3 months after intervention)

Abbreviations: ISP, Internet‐based Support Program.

**Figure 3:**
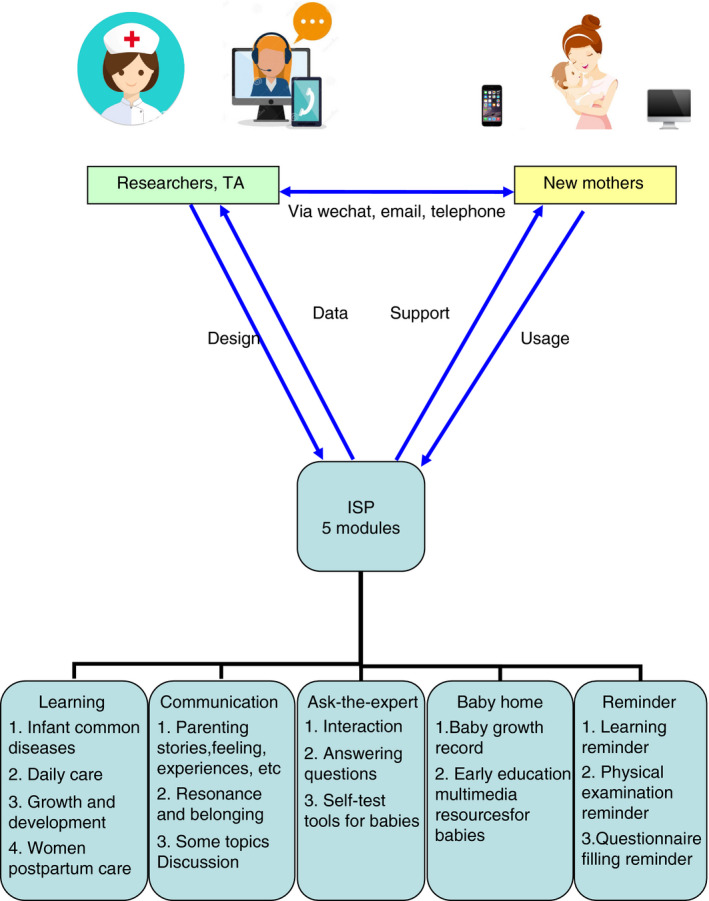
ISP program and its five modules. Note: TA, Technical assistants; ISP, Internet‐based support program [Colour figure can be viewed at wileyonlinelibrary.com]

ISP (Figure 3) has a fixed structure and lasts approximately 3 months. Five modules are designed to meet learning needs, communication needs, emotional needs, and presentation needs for primiparous women. Module 1 is learning forum of parenting knowledge and skills showed by multimedia resources, such as text, picture, and video. The contents include: (1) Prevention, judgment, and intervention of common diseases for infants, such as eczema, pathological jaundice, diarrhoea, constipation, cough, allergy, fever, trachea foreign body, pneumonia, and thrush; (2) Daily care: including dressing, changing diapers, bathing, dietary feeding, umbilical cord care, sleep problems, urinary and faecal problems, prevention of accidental injury, and other safety issues, etc; (3) Growth and development: including normal and abnormal performance of newborn babies, e.g., motor ability, language ability, socialized emotion, and socialized development; baby touch and parent–child interaction; and (4) Postpartum care: including coping with postpartum physical and mental changes, postpartum exercise for women, etc. Module 2 is communication forum where new mothers can tell their parenting stories, exchange parenting experiences, seek resonance, and belonging. Women are also invited to discuss some topics in this forum such as the transition to motherhood, Chinese traditional practice of “Doing the month”, obstacles to communication, potential interpersonal conflict after childbirth, and skills for resolving interpersonal conflicts. Ask‐the‐experts forum is module 3 and health professionals can interact with primiparous women, answer their parenting question posted by individuals within 24 hr. Some self‐test tools for baby also are included to help new mother to get some useful information, such as Bayley Scales of Infant and Toddler Development (BSID), growth and development curve for Chinese children, baby immunization query, etc. Baby home forum is module 4 where mothers can record and share their babies’ growth and their feelings. Some early education multimedia resources likewise are in this forum, such as children's songs, animation, story, and ancient poetry. Module 5 is reminder forum, including ISP learning reminder, postpartum physical examination reminder, and questionnaire filling reminder.

The researchers will teach women in the intervention group how to log in and use each module of ISP by mobile phone or computer. The original username and password of women will be setup by the researchers; and can be changed by women later. The frequency and duration of the logins will be monitored to evaluate the women's adherence. Technical assistance will be available on the weekdays by telephone or email.

#### 3.2.6 Outcome measurements

##### Primary outcomes

###### MSE

*MSE* will be evaluated by the Self‐efficacy in Infant Care Scale (SICS) (Prasopkittikun & Tilokskulchai, 2010). The SICS is a 46‐item scale comprising of four dimensions. Each item indicates one parenting task rated from 0 to 100 points. A higher SICS score means a higher level of *MSE*. The SICS has demonstrated good psychometric properties. The reported internal consistency was 0.96 for the total scale and ranged from 0.86 to 0.96 for its dimensions (Prasopkittikun & Tilokskulchai, 2010). In terms of Chinese version SICS, its Content Validity Index (CVI) was 0.98; and the internal consistency was 0.95 for the scale and was in range 0.80–0.93 for its dimensions (Zang & Sheng, 2010).

###### PPD

PPD will be assessed using the Edinburgh Postnatal Depression Scale (EPDS) (Cox, Holden, & Sagovsky, 1987). The scale is a 10‐item 4‐point Likert instrument to detect PPD. The self‐report scale's score ranges between 0 and 30 and the higher score indicates the worse health status women have. The EPDS has demonstrated good reliability and validity. The Cronbach's alpha coefficient of the Chinese version EPDS was 0.87; and the concurrent validity with the Beck Depression Inventory was 0.79 (Wang et al., 2009).

###### Social support

Postnatal social support will be measured using the Postpartum Social Support Scale (PSSS), developed for Chinese women to measure their perceived social support after childbirth (Lu & Zheng, 2001). The scale is a 20‐item four Likert self‐report tool and its score is in range 0–60 overall. The higher score equates to the more social support a mother receives. The Cronbach's alpha coefficient of PSSS was 0.89 and the content validity of this tool was 0.90 (Lu & Zheng, 2001).

##### Secondary outcomes

###### Satisfaction with healthcare

Women's satisfaction with health care after childbirth will be assessed with a 3‐item questionnaire developed by the researcher. For instance, “Overall, how satisfied you are with the healthcare?”, “Please give your reasons”, and “Please give your suggestions”.

##### Other outcomes

###### Social‐demographic and clinical data

The collected data comprise the maternal age, marital status, educational level, occupation, family income, mode of birth, baby gender, baby health, and baby fussiness via women's self‐report.

###### Internet usage data for the ISP

Internet use data, such as the frequency and duration of logins and the website activity of each module of the ISP, will be recorded and evaluated by a tracking system from the website.

#### 3.2.7 Data collection procedure

The baseline assessment will be conducted by the researchers and every participant will be asked to complete the SICS, EPDS, PSSS, and social‐demographic and clinical data in the obstetric wards. Post‐test 1 will be carried out immediately after the intervention and Post‐test 2 will be conducted on 3 months after the intervention. At the two time points of Post‐tests 1 and 2, the questionnaires comprised of SICS, EPDS, PSSS, and healthcare satisfaction will be sent to participants by wechat or email; and the completed questionnaires will be returned to the researchers by wechat or email. To improve the response rate, a kindly telephone or wechat reminder will be given to participants before and after one week of the two time points, respectively.

#### 3.2.8 Data analysis

Data will be analysed using the Statistical Package for Social Sciences (SPSS). An intention‐to‐treat analysis (ITT) will be adopted to manage missing data. Descriptive statistics were conducted to describe the sociodemographic and clinical characteristics of primiparous women by means and standard deviations (SDs); frequencies and proportions. The chi‐square (χ2) and the independent sample *t* test will be used to detect any significant difference between the study and control groups on the baseline variables. Adjusted for possible confounding factors of sociodemographic variables, the repeated measures triply MANCOVA will be conducted to determine whether the intervention of ISP is effective in enhancing *MSE* and social support and for reducing PPD across the three time points of data collection (baseline, post‐test 1, and post‐test 2). The independent *t* test will be used to compare how satisfied the intervention group and the control group are with the health care that they received after childbirth on the two time points of immediately after the intervention and 12 weeks after intervention.

### 3.3 Ethical considerations

Research ethical committee approval is obtained by the Research Ethics Committee of Health Science Center in a University of China (approval number 2,020,011). The registration number with the Chinese Clinical Trial Registry is ChiCTR2000033154. This study will adhere to ethical standards for the whole procedure. There is no potential risk or harm by participating in this program. Women will not be deprived of any treatment and routine care. The written informed consent will be obtained from every participant before data collection. Women will be informed of freedom to withdraw at any time and are assured of anonymity by using special code numbers to identify themselves. All of the collected data will be kept anonymously and confidentially.

### 3.4 Validity and reliability

This study adopts the rigorous research design, such as the sound theoretical framework, a RCT design with a good representative and predetermined sample, the use of instruments with a high validity and reliability, and statistically scientific analysis, which can be seen to reduce bias effectively and enhance the generalizability of research results beyond the target population. Moreover, outcome assessors and data analysts of the research will be blinded after assignment to interventions to reduce the biases in evaluation of the effects of the intervention.

## 4 DISCUSSION

To my knowledge, this study will be the first RCT by internet on parenting outcomes for Chinese primiparous women. If the ISP program has positive outcomes, it will contribute to the scientific and practical knowledge of nursing interventions to support primiparous women on parenting outcomes; and could become the routine health care for health professionals to enhance parenting ability and mental well‐being of new mothers.

### 4.1 Limitations

Some limitations need to be noted in the research. First, due to the nature of the research, blinding of researchers during all the research process is not possible and it may lead to the potential biases. Secondly, the participants are limited to those who are willing to participate in this study via internet. The results might not be generalized to those who refused to participate in the research or those who are not available to the internet. Thirdly, the longer‐term intervention will be conducted in this study and some participants may be loss during the follow‐up that can decrease the study power. To overcome the problems, a 30% attrition rate is considered in the sample size calculation and an ITT analysis will be performed in the research.

## 5 CONCLUSION

This research will contribute to evidence on the effectiveness of using internet platform to support women after childbirth. Such knowledge could help to advance research regarding the use of internet‐based intervention methods to improve women's *MSE*, social support, satisfaction, and to alleviate depression symptoms. The ISP with expected outcomes could be implemented in clinical practice to enhance parenting ability and mental well‐being for Chinese new mothers. If the ISP is effective, it also could provide the new intervention program for the further research. Furthermore, the knowledge gained from this study could be used to plan a culturally appropriate ISP for other women, such as first‐time mothers from different countries, single mothers, multiparas, and those with complicated pregnancy.

## CONFLICT OF INTEREST

None.

## Author Contributions

Study design: XJZ, QW; data collection: XJZ; LLH, QYF, YZ, KL, XLL, YZ; data analysis: XJZ; LLH, QYF, YZ, KL, XLL, YZ; QW; manuscript preparation: XJZ; and manuscript revision: XJZ, QW.

### Peer Review

The peer review history for this article is available at https://publons.com/publon/10.1111/jan.14517.
